# Metabolism and regulation of chlorophyll and carotenoids in tomato fruits

**DOI:** 10.1093/hr/uhag084

**Published:** 2026-03-04

**Authors:** Weiqing Zhang, Ying Xie, Ying Yuan, Qian Long, Zhiyong Shao, Jirong Zheng, XueJuan Ru, Jia Luo, Guanghui Pan, Olubukola Oluranti Babalola, Wei Deng

**Affiliations:** Key Laboratory of Plant Hormones and Development Regulation of Chongqing, School of Life Sciences, Chongqing University, Chongqing 400044, China; Center of Plant Functional Genomics and Synthetic Biology, Institute of Advanced Interdisciplinary Studies, Chongqing University, Chongqing 400044, China; Key Laboratory of Plant Hormones and Development Regulation of Chongqing, School of Life Sciences, Chongqing University, Chongqing 400044, China; Key Laboratory of Plant Hormones and Development Regulation of Chongqing, School of Life Sciences, Chongqing University, Chongqing 400044, China; Key Laboratory of Plant Hormones and Development Regulation of Chongqing, School of Life Sciences, Chongqing University, Chongqing 400044, China; Hangzhou Academy of Agricultural Sciences, Hangzhou 310024, China; Chongqing Academy of Agricultural Sciences, Chonhqing 401329, China; Chongqing Academy of Agricultural Sciences, Chonhqing 401329, China; Chongqing Academy of Agricultural Sciences, Chonhqing 401329, China; Chongqing Academy of Agricultural Sciences, Chonhqing 401329, China; Food Security and Safety Focus Area, Faculty of Natural and Agricultural Sciences, North-West University, Private Bag X2046, Mmabatho 2735, South Africa; Key Laboratory of Plant Hormones and Development Regulation of Chongqing, School of Life Sciences, Chongqing University, Chongqing 400044, China; Center of Plant Functional Genomics and Synthetic Biology, Institute of Advanced Interdisciplinary Studies, Chongqing University, Chongqing 400044, China

## Abstract

Tomato (*Solanum lycopersicum*) is a nutrient-rich and flavorful vegetable, ranking among the most consumed globally. In recent years, consumers have increasingly demanded high-quality tomatoes, prompting the extensive research into the key factors and relevant molecular mechanisms regulating the formation of fruit quality. Coloration is a crucial aspect determining the appearance quality of tomato fruits and directly affecting their commercial value. This coloration is intrinsically linked to the composition and abundance of special chemical compounds in fruits, particularly chlorophyll and carotenoids. Chlorophyll is the predominant pigment accumulated in the early stages of fruit development and plays a vital role in photosynthesis. As the fruit ripens, chlorophyll undergoes gradual degradation, while carotenoids are abundantly synthesized, resulting in a striking color transition from green to red. Chlorophyll and carotenoids are essential natural pigments and antioxidants that are indispensable for both coloration and nutritional value of tomato fruits. This review presents a comprehensive overview of the metabolic pathways and regulatory mechanisms of these metabolites, aiming to provide novel insights and strategies for improving tomato quality to meet the growing consumer demand for fruits with appealing coloration and enhanced nutrients.

## Introduction

Tomato (*Solanum lycopersicum*) is a globally important crop and a premier model for fleshy fruit research, owing to its nutritional value, genetic tractability, and well-annotated genome [1, 2]. This makes it an ideal organism for investigating the genetic and molecular basis of fruit development, ripening, and quality formation [3]. Fruit quality, which reflects the integrated outcome of growth and development, is a critical determinant of commercial value [4]. Consequently, improving tomato fruit quality represents a key agricultural and scientific goal, with implications for both market acceptance and research on fleshy fruits.

Preharvest fruit quality encompasses appearance, organoleptic properties, and nutritional content [5, 6]. Coloration, a primary visual trait, reflects ontogenetic stages and metabolic activities during development and ripening [7]. Immature green fruits accumulate chlorophyll, which supports photosynthesis and early development. During ripening, chlorophyll progressively degrades while carotenoids accumulate, leading to the characteristic red coloration of ripe tomatoes [8]. The levels and composition of chlorophyll, carotenoids (notably the proportion of lycopene and β-carotene), and other pigments like anthocyanins determine the final fruit color, providing key targets for breeding varieties with diverse pigmentation [9]. These pigments are also nutritionally vital [10]. Chlorophyll supports photosynthetic activity in developing fruit, which promotes carbohydrate accumulation and ultimately influences sugar levels at ripeness [11–13]. Delaying chlorophyll degradation can extend the photosynthetic period, enhancing carbon assimilation and nutrient content [14]. Carotenoids act as antioxidants and as precursors for phytohormones and volatiles, thereby improving fruit quality and potential health benefits [15].

Given the central role of chlorophyll and carotenoids in coloration and nutrition, a deep understanding of their metabolic homeostasis is essential for quality improvement. Although their metabolic pathways have been widely studied, the regulatory mechanisms warrant further synthesis. This review summarizes current knowledge on the metabolism and regulation of chlorophyll and carotenoids in tomato fruit, with the aim of providing a cohesive view of color formation and strategies for quality enhancement.

## Metabolism of chlorophyll and carotenoids in tomato fruit

### Chlorophyll biosynthesis

**Figure 1:**
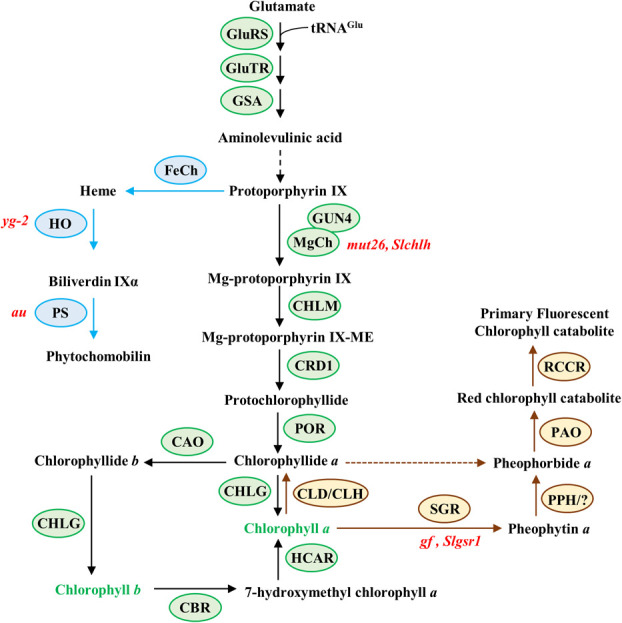
Biosynthetic and degradative pathways of chlorophyll in tomato. Enzymes involved in chlorophyll biosynthesis, degradation, and heme biosynthesis pathways are highlighted within green, yellow, and blue circles, respectively. Tomato mutants of corresponding genes are indicated in red. Dark-dotted arrow represents multiple enzymatic steps not shown in detail, while the yellow-dotted arrow marks a step that remains unelucidated. A question mark denotes the unidentified enzyme responsible for converting pheophorbide *a* to red chlorophyll catabolites in tomato fruits. GluRS, glutamyl-tRNA synthetase; GluTR, glutamyl-tRNA reductase; GSA, glutamate-1-semialdehyde 2,1-aminomutase; FeCh, ferrochelatase; HO, heme oxygenase; PS, phytochromobilin synthase; MgCh, magnesium chelatase (a multisubunit complex composed of CHLH, CHLI and CHLD); GUN4, Genomes Uncouple 4 (an activator of MgCh); CHLM, Mg-protoporphyrin IX methyltransferase; CRD1, Mg-protoporphyrin IX monomethylester cyclase; POR, NADPH: protochlorophyllide oxidoreductase; CAO, chlorophyllide *a* oxygenase; CHLG, chlorophyll synthase; HCAR, 7-hydroxymethylchlorophyll *a* reductase; CLD, chlorophyll dephytylase; SGR, stay green, the Mg-dechelatase; PAO, pheophorbide a monooxygenase; RCCR, red chlorophyll catabolite reductase. *yg-2* (*yellow-green-2*) and *au* (*aurea*) mutants exhibit phytochrome chromophore deficiency due to lesions in HO and PS, respectively. *mut26*, a missense mutant of *SlCHLH*. *Slchlh*, CRISPR-Cas9 gene edited mutant of *SlCHLH*. *gf*, *green flesh* mutant, carrying a single nucleotide substitution in the coding domain sequence of *SlSGR1*. *Slgsr1*, CRISPR-Cas9-generated knockout mutant of *SlSGR1*.

Chlorophyll, a magnesium-containing porphyrin, is synthesized in chloroplasts via the tetrapyrrole biosynthesis pathway [16]. In higher plants, this pathway initiates from glutamate and proceeds through key intermediates, including protoporphyrin IX (PPIX), a common precursor for both heme and chlorophyll (Fig. 1). Within the chlorophyll synthesis pathway, PPIX is converted into chlorophyll *a* and *b* through sequential enzymatic steps [17]. Notably, chlorophyll *b* can be reconverted to chlorophyll *a* via the chlorophyll cycle, which involves the coordinated actions of chlorophyll *b* reductase (CBR) and 7-hydroxymethyl chlorophyll *a* reductase (HCAR) [18]. Chlorophyll *a* and *b* are the primary types of chlorophyll found in higher plants, playing essential roles in photosynthesis through light energy absorption.

Chlorophyll biosynthesis is highly conserved and vital for photosynthesis, stress responses, and development [19]. In tomato, chlorophyll biosynthesis genes are active in leaves and immature green fruit [20–22]. Functional studies of tomato mutants, including those with the *SlCHLH* (*mut26*) missense mutation and fruit-specific suppression of *glutamate 1-semialdehyde aminotransferase* (*GSA*), highlight the importance of chlorophyll biosynthesis enzymes in fruit development [22, 23]. Notably, the subunit of Mg-chelatase SlCHLH not only participates in chlorophyll production but also serves as chloroplast retrograde signal to promote ripening and ethylene biosynthesis [22]. This dual functionality exemplifies how chlorophyll biosynthetic enzymes can pleiotropically influence fruit quality beyond pigmentation alone. While many enzymes in the chlorophyll synthesis pathway are known to act as chloroplast retrograde signals to regulate nuclear gene expression in plants [16], their specific functions in tomato fruit remain largely uncharacterized.

### Chlorophyll degradation

As a crucial physiological counterpart to its synthesis, chlorophyll degradation is a fundamental process in plants that occurs throughout growth and developmental stages. The major pathway is initiated by Mg dechelation of chlorophyll *a*, a reaction catalyzed by STAY-GREEN (SGR) proteins that produces pheophytin *a* [24]. Pheophytin pheophorbide hydrolase (PPH) then converts pheophytin *a* to pheophorbide *a*, which enters the core pheophorbide a monooxygenase (PAO)-mediated pathway, yielding red chlorophyll catabolites (Fig. 1) [25, 26]. An alternative route involves initial dephytylation of chlorophyll *a* by chlorophyllase (CLH) or chlorophyll dephytylase (CLD) to form chlorophyllide *a*, which is subsequently converted to pheophorbide *a* [27]. The PAO pathway is well characterized during leaf senescence and is also operational in fruits, where chlorophyll breakdown facilitates color change and often reveals underlying carotenoids, as seen during tomato ripening [28].

Current understanding of chlorophyll degradation in fruits centers on SGR proteins, which function as Mg-dechelatases [18, 26]. Tomato possesses three *SGR* genes: *SlSGR1* and *SlSGR2* are highly expressed during fruit ripening, while *SlSGRL* is leaf-specific and primarily associated with senescence [29, 30]. SlSGR1 is regarded as the principal Mg dechelatase driving chlorophyll degradation during ripening. Both the classical *green flesh* (*gf*) mutant (with an amino acid substitution in SlSGR1) and CRISPR-engineered *Slsgr1* mutant exhibit impaired chlorophyll breakdown, leading to delayed color transition and ripe fruits with a characteristic muddy-brown appearance and persistent chlorophyll [31, 32]. *SlSGR2* is responsive to cadmium and salt stresses, implying a role in abiotic stress response [29, 33], but its specific function in fruit pigmentation and stress signaling remains unclear.

PPH is another key enzyme, being the sole identified dephytylase in the core chlorophyll catabolic pathway [34]. Although evolutionarily conserved in higher plants, PPH appears dispensable in certain tissues such as fruits and seeds [27]. In tomato, *SlPPH* is highly expressed during leaf senescence and fruit ripening. Its suppression results in a stay-green phenotype in leaves, resembling *gf* mutants. However, in fruits, disruption of *SlPPH* only temporarily delays chlorophyll loss and does not produce a persistent green phenotype, indicating its contributory but non-essential role during ripening [35]. This suggests that while SlPPH is integral to leaf chlorophyll catabolism, its activity in fruits likely operates in coordination with other hydrolases or through alternative degradation routes [27, 35]. SlPPH also influences carbon metabolism and nutraceutical content in fruits, extending its role beyond pigment metabolism [36]. The specific dephytylase catalyzing chlorophyll degradation in tomato fruit has yet to be identified, and its characterization is crucial for a full understanding of this process.

Interestingly, both CLD and CLH exhibit dephytylation activity. *CLD* is predominantly expressed in green tissues, where it supports chlorophyll turnover under steady-state conditions [37, 38]. Although CLH has traditionally been considered the initial enzyme in chlorophyll degradation, studies in *Arabidopsis* suggest that PPH, rather than CLH, catalyzes the key dephytylation step during leaf senescence, leaving CLH's exact role in this process unclear [39]. Recent evidence indicates that *Arabidopsis* CLHs are involved in photosystem II repair, mitigate photodamage in young leaves, and participate in responses to abiotic and biotic stress [40–42]. In specialized tissues, CLH contributes to chlorophyll degradation. For instance, CLH1 promotes petal degreening in *Paeonia suffruticosa* [43], and citrus CLH facilitates fruit color transition, with its expression being ethylene-responsive and inversely correlated with chlorophyll levels [39, 44]. Tomatoes have four CLHs. While *SlCLH2* and *SlCLH3* transcripts are low or almost undetectable in fruits, *SlCLH1* expression peaks at mature green stage, and *SlCLH4* exhibits dynamic expression during ripening [45]. Despite these expression patterns, the precise functions of SlCLHs in chlorophyll catabolism during tomato fruit ripening remain poorly understood. Thus, the complete enzymatic network controlling chlorophyll degradation in tomato fruit is yet to be fully elucidated and remains an active research focus.

### Carotenoid biosynthesis

**Figure 2:**
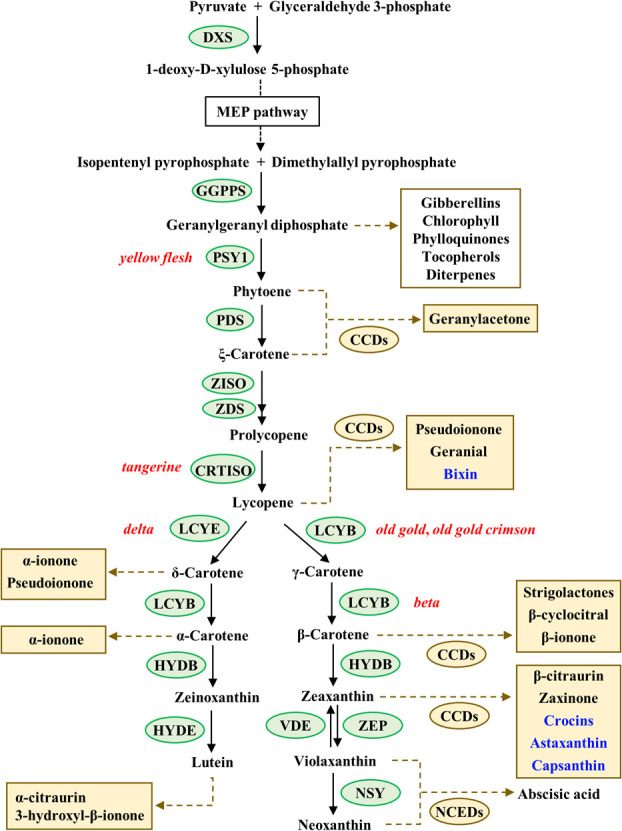
Biosynthetic and degradative pathways of carotenoids in tomato. Enzymes involved in carotenoid biosynthesis and degradation are highlighted within green and yellow circles, respectively. Tomato mutants of these enzymes are indicated in red. Yellow-dotted arrows represent multiple enzymatic steps catalyzed by the putative CCDs or NCEDs to form apocarotenoids. Compounds in yellow boxes denote apocarotenoids derived from carotenoid degradation, while those highlighted in blue within these boxes represent specific natural products that have been or are planned to be genetically engineered into tomato fruits. DXS, 1-deoxy-D-xylulose 5-phosphate synthase; LCYB, lycopene β-cyclase (also commonly designated as CYCB); MHO, 6-methyl-5-hepten-2-one. *Yellow flesh*, the mutant of *SlPSY1*, produces yellow fruits. *Tangerine*, the mutant of *SlCRTISO*, accumulates prolycopene and develops orange fruits. *Old gold/old gold crimson*, loss-of-function mutant of *SlCYCB*, produces deep red fruits with elevated lycopene levels. *Beta*, gain-of-function mutant of *SlCYCB*, yields orange fruits with reduced lycopene content. *Delta*, loss-of-function mutant of *SlLCYE*, accumulates lycopene and exhibits red coloration.

Carotenoids are a diverse group of natural pigments that perform essential biological functions and encompass over 800 known compounds, spanning a color spectrum from colorless to yellow, orange, and red [46]. These isoprenoids are synthesized in plant plastids through the methylerythritol 4-phosphate (MEP) pathway, which begins with the condensation of pyruvate and glyceraldehyde 3-phosphate catalyzed by 1-deoxy-d-xylulose 5-phosphate synthase (DXS) and ultimately yields the universal five-carbon precursors, isopentenyl pyrophosphate (IPP) and dimethylallyl pyrophosphate (DMAPP) [47, 48]. These precursors are condensed by geranylgeranyl diphosphate synthase (GGPPS) to form geranylgeranyl diphosphate (GGPP), a central intermediate not only for carotenoids but also for chlorophyll, gibberellins, and diterpenoids (Fig. 2) [49, 50]. Carotenoid biosynthesis commits with phytoene synthase (PSY), which condenses two GGPP into phytoene [51]. Phytoene is typically converted to lycopene through sequential desaturation and isomerization reactions catalyzed by phytoene desaturase (PDS), ζ-carotene desaturase (ZDS), ζ-carotene isomerase (ZISO), and carotenoid isomerase (CRTISO) [47]. Lycopene, an acyclic carotene abundant in red fruits, can be cyclized by lycopene ε-cyclase (LCYE) and lycopene β-cyclase (LCYB, also commonly designated as CYCB) to produce α-carotene and β-carotene, respectively [49]. α-carotene is further hydroxylated by carotenoid β-ring hydroxylase (HYDB) and carotenoid ε-ring hydroxylase (HYDE) to yield lutein, while β-carotene is hydroxylated by HYDB to form zeaxanthin [52]. Zeaxanthin is then converted successively to violaxanthin and neoxanthin by zeaxanthin epoxidase (ZEP) and neoxanthin synthase (NSY) [53]. Notably, violaxanthin de-epoxidase (VDE) can convert violaxanthin back to zeaxanthin [54].

The carotenoid biosynthetic pathway and most of its key enzymes in tomato fruit have been well characterized. Genetic manipulation of these enzymes provides a crucial strategy for engineering specific carotenoid profiles [55]. *SlPSY1* mutant *yellow-flesh* exhibits substantially reduced carotenoid content, particularly lycopene and its derivatives, resulting in yellowish fruits [56]. *SlLCYE* variant possessing missense mutation (G/3378/T) increases lycopene and total carotenoids in ripe fruits [57]. Simultaneous silencing of *SlLCYB1* and *SlLCYB2* induces severe mottled photobleaching in leaves and fruits, whereas *SlLCYB2* overexpression develops orange fruits and alters carotenoid composition [58]. Additionally, *SlCYCB* mutants, arising from spontaneous *old-gold* or *old-gold crimson* mutations, the A949G mutation, or CRISPR-Cas9–mediated genetic alterations, produce deep red fruits with elevated lycopene levels [59–61]. These studies underscore the potential of targeted genetic modifications to precisely engineer carotenoid profiles and fruit color in tomato.

### Carotenoid degradation

Carotenoids contain a conjugated double-bond system that renders them susceptible to oxidative cleavage by carotenoid cleavage oxygenases (CCOs), generating apocarotenoids, such as β-cyclocitral, β-ionone, and abscisic acid (ABA) (Fig. 2) [62, 63]. CCOs comprise carotenoid cleavage dioxygenases (CCDs) and 9-cis-epoxycarotenoid dioxygenases (NCEDs), which are widely distributed in higher plants with species-specific gene numbers [62]. For example, *Arabidopsis* has four CCDs (CCD1/4/7/8) and five NCEDs (NCED2/3/5/6/9) [62, 64], while tomato harbors seven CCDs (SlCCD1a/1b/4a/4b/7/8 and SlCCD-like) and eleven NCEDs (SlNCED1–11) [65, 66]. CCD1 and CCD4 cleave diverse double bonds in carotenoids such as β-carotene and zeaxanthin, producing volatile compounds like β-ionone and pseudoionone that contribute to fruit flavor and aroma [62, 67]. CCD7 and CCD8 are indispensable for strigolactones (SLs) biosynthesis from β-carotene [63], whereas NCEDs catalyze the cleavage of violaxanthin and neoxanthin to form xanthoxin, the precursor of ABA [68]. Apocarotenoids function as hormones, volatiles, and signaling molecules, playing key roles in plant development under tight metabolic control [69, 70].

In tomato, *SlCCD1* knockdown reduces levels of β-ionone and geranylacetone in fruits, confirming its role in flavor-volatile production [71, 72]. SlCCD4b preferentially cleaves β-carotene and ζ-carotene, promoting geranylacetone and β-ionone production. Its overexpression alters pigment composition, leading to dark-orange fruits with elevated phytoene, ζ-carotene, and neurosporene but reduced lycopene, β-carotene, and lutein [73]. SlCCD7 and SlCCD8, together with DWARF27 (SlDR27), convert β-carotene to carlactone, the SL precursor [74–76]. *SlNCED1* is prominently expressed in fruits, particularly during the ripening stage and is accountable for ABA production in these fruits [77, 78]. Its specific interference in fruits leads to deep red coloration, enhanced lycopene and β-carotene accumulation, and decreased ABA levels [78, 79]. These findings have spurred breeding and metabolic engineering approaches aimed at enhancing tomato pigmentation and nutritional carotenoid content (e.g. lycopene, carotene, lutein, zeaxanthin) [80–82].

Notably, carotenoids are precursors for high-value colorants and nutraceuticals, such as bixin, capsanthin, crocin, and astaxanthin, typically obtained from specialized plants [83]. Carotenoids-rich tomatoes hold immense potential as chassis cells in synthetic biology for producing diverse natural products and enhancing fruit value. Engineered tomatoes accumulating crocins show strong antioxidant activity and protect neuroblastoma cells from oxidative stress [84]. Astaxanthin-producing lines also exhibit higher lycopene and delayed over-ripening [85]. Tomatoes engineered to synthesize ketocarotenoids have been successfully tested in aquaculture [86]. Furthermore, tomato has been proposed as a suitable platform for producing carotenoid derivatives like bixin and capsanthin, though this remains to be fully achieved [87, 88]. Therefore, a thorough understanding of tomato carotenoid metabolism is crucial for tailoring fruit color and nutritional quality, positioning tomato as a key source of high-value plant-derived products.

## Regulation of chlorophyll and carotenoid metabolism

### Transcriptional regulation of chlorophyll metabolism

Chlorophyll biosynthesis occurs in chloroplasts, whose development and function are tightly regulated by a network of transcription factors (TFs) [89]. Golden 2-like (GLK) proteins are key positive regulators that promote chloroplast development and chlorophyll biosynthesis by activating genes associated with chloroplast development and photosynthesis [90]. Tomatoes possess two *GLK* genes: *SlGLK1* is predominantly expressed in leaves, while *SlGLK2* shows a primary expression in fruits and is encoded by *uniform ripening* (*u*) locus [91]. *SlGLK2* overexpression increases chloroplast quantity and size, leading to uniformly dark-green fruits with green shoulders, a phenotype dependent on its gradient expression across the fruit [91, 92]. It also promotes the accumulation of chlorophyll, sugar, tocopherol, and carotenoids in fruits by modulating related metabolic genes, making *SlGLK2* a promising target for improving tomato quality [93, 94]. Although *SlGLK1* is primarily leaf-expressed, its overexpression similarly elevates fruit chlorophyll, indicating functional overlap with *SlGLK2* [91].

**Figure 3:**
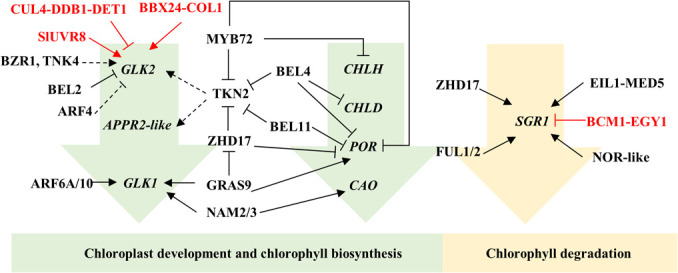
Regulation of chlorophyll metabolism in tomato fruit. Genes involved in chloroplast development and chlorophyll metabolism are shown in italics, and transcription factors directly targeting these genes are highlighted in non-italics. Dark arrows and short lines indicate positive and negative regulation by TFs, respectively. Red arrows and short lines signify that the stability of enzymes associated with chloroplast development and chlorophyll metabolism is activated and suppressed by specific regulators (marked in red) through protein–protein interactions, respectively. APPR2-like, Arabidopsis Pseudo Response Regulator 2-Like; BBX24, B-Box 24; BCM1, Balance of Chlorophyll Metabolism 1; BEL, Bel1-like homeodomain; BZR1, Brassinazole Resistant 1; CHLH, magnesium chelatase H subunit; CHLD, magnesium chelatase D subunit; CUL4, Cullin 4; COL1, Constans-Like 1; DDB1, UV-damaged DNA binding protein; DET1, de-etiolated 1; EGY1, Yellow Green 1; EIL1, Ethylene insensitive 3 like/EIN3-like 1; FUL, FRUITFULL; GLK, Golden 2-like; GRAS9, Gibberellic-acid insensitive (GAI), Repressor of GAI, and Scarecrow 9; MYB72, myeloblastosis 72; MED5, Mediator complex 5; NAM, NO Apical Meristem; NOR-like, Non-ripening like; ZHD17, Zinc-finger homeodomain 17.

Given their central roles in chloroplast development and chlorophyll accumulation in tomato fruits, *SlGLK1* and *SlGLK2* are regulated by multiple upstream factors (Fig. 3). *SlGLK2* is repressed by Bel1-like homeodomain 2 (SlBEL2), which binds to its promoter and interacts with the protein to inhibit its transcriptional activity [95]. The class I Knotted-like homeodomain TFs SlTKN2 and SlTKN4 promote fruit chloroplast development and positively regulate *SlGLK2* [96]. Auxin also regulates chlorophyll biosynthesis through auxin response factors (ARFs). SlARF4 acts as a negative regulator, likely by binding to the *SlGLK2* promoter to suppress its expression, while reduced *SlARF4* expression leads to dark-green fruits with elevated chlorophyll and *SlGLK1* transcript levels [93, 97]. Conversely, SlARF6A and SlARF10 enhance chlorophyll accumulation in fruits by directly activating the *SlGLK1* promoter and upregulating genes involved in chloroplast development and chlorophyll metabolism [98, 99]. Additionally, overexpression of *Brassinazole Resistant 1* (*SlBZR1*) produces dark-green shoulder phenotypes similar to *SlGLK2*-mediated effects, accompanied by strong *SlGLK2* up-regulation [100]. However, direct binding of SlTKN2, SlTKN4, and SlARF4 to the *SlGLK2* promoter remains to be experimentally confirmed.

SlAPRR2-Like (Arabidopsis Pseudo Response Regulator 2-Like) protein is also essential for fruit chloroplast development and pigment accumulation. It is transcriptionally activated by SlTKN2, and its overexpression results in dark-green fruits characterized by enlarged plastids and elevated chlorophyll levels (Fig. 3) [96, 101]. Notably, SlAPRR2-Like functions independently of SlGLK2, with no mutual influence on their expression. However, its precise regulatory mechanism in fruit chloroplast development remains unclear. Additionally, multiple TFs directly target key genes in chlorophyll biosynthesis and chloroplast development (Fig. 3). SlBEL11 negatively regulates chloroplast development and chlorophyll accumulation in immature fruits by suppressing the promoter activity of *SlTNK2*, *SlCAB*, and *SlPOR* [20]. SlBEL4 represses *SlCHLD*, *SlPOR*, *SlTNK2* and *SlCAB-1C/3B*, leading to reduced plastid size, number, and chlorophyll content in fruits [102]. SlMYB72 directly inhibits *SlCHLH*, *SlPOR* and *SlTKN2* through promoter binding [103]. Interacting with SlMYB72, SlZHD17 suppresses *SlPORB* and *SlTKN2* while promoting *SlSGR1* expression in fruits [104]. *SlZHD17* itself is transcriptionally upregulated by SlGRAS9, which also directly suppresses *SlPORB* and *SlGLK1* to modulate chlorophyll synthesis [21]. Moreover, the NAC (NAM, ATAF1/2, CUC2) TFs SlNAM2 and SlNAM3 activate *SlCAO2*, *SlPORC*, *SlGLK1*, and *SlCAB11*, thereby promoting chloroplast development and delaying fruit ripening, ultimately affecting fruit quality [105].

Regulating chlorophyll degradation is essential for maintaining chlorophyll homeostasis in tomato fruits. As some enzymes in the chlorophyll degradation pathway during fruit ripening remain experimentally uncharacterized, with only SlSGR1 being fully confirmed, research on chlorophyll breakdown regulation has primarily focused on this gene. Beyond its role in chlorophyll catabolism, SlSGR1 also regulates carotenoid content, thereby influencing fruit color transition through coordinated modulation of chlorophyll degradation and carotenoid biosynthesis [31, 106]. Consequently, SlSGR1 acts as a central regulator of ripening-associated color change and is directly activated by key ripening-related TFs, including Ripening Inhibitor (RIN), Non-Ripening (NOR), FRUITFULL1/2 (FUL1/2), SlZHD17, and Ethylene insensitive 3 like/EIN3-like (EIL1)-Mediator complex (MED5) complex (Fig. 3) [104, 107–110]. Chlorophyll degradation is a critical trigger for fruit ripening and responds to both intracellular and environmental signals. Identifying structural genes involved in fruit chlorophyll breakdown, as well as novel regulators that control their expression directly or indirectly, remains essential for delineating the fruit-specific chlorophyll degradation pathway.

### Transcriptional regulation of carotenoid metabolism

**Figure 4:**
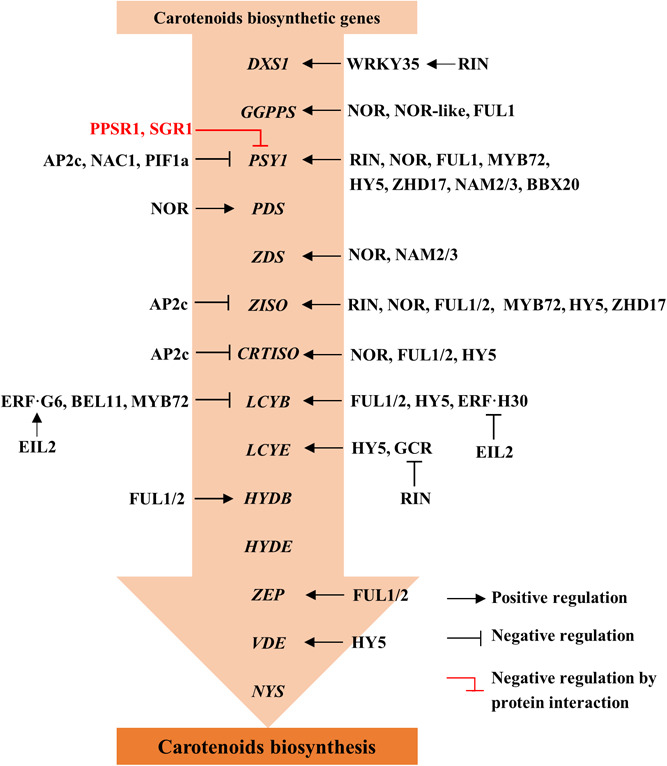
Regulation of carotenoid biosynthesis in tomato fruits. Genes involved in carotenoid biosynthesis are shown in italics, and transcription factors (TFs) directly targeting these genes are highlighted in non-italics. Dark arrows indicate positive regulation by TFs, while short lines denote negative regulation. The red short line signifies that the activity and stability of carotenoid biosynthetic enzymes are inhibited by specific regulators (marked in red) through protein–protein interactions. AP2, APETALA2; GCR, G2-like Carotenoid Regulator; HY5, hypocotyl 5; NOR, non-ripening; PIF1a, phytochrome interacting factor 1a; PPSR1, Plastid Protein Sensing RING E3 ligase 1; RIN, ripening inhibitor; NAM, no apical meristem.

As carotenoids predominantly accumulate in ripening fruits, their biosynthetic genes are largely controlled by ripening-related factors, such as APETALA2 (AP2), colorless non-ripening (CNR), FUL1/2, NOR, and RIN (Fig. 4) [111]. Disruption of these regulators substantially alters carotenoid levels and compositions by directly or indirectly modulating carotenoid metabolic genes, leading to distinct fruit coloration [111]. SlRIN directly activates the promoters of *SlPSY1*, *SlZISO*, and *SlCRTSO* [107, 112] and also induces *SlWRKY35* expression, which in turn upregulates *SlDXS1* and enhances carotenoid content [113]. The GARP G2-like TF SlGCR (G2-like Carotenoid Regulator) promotes lutein biosynthesis by directly stimulating *SlLCYE* expression, though it is itself repressed by SlRIN [114]. SlNOR-like directly activates *SlSGR1* and the fruit-specific *SlGGPPS2* [109, 115], whereas SlNOR exhibits specific affinity for the promoters of *SlGGPPS1*, *SlPSY1*, *SlPDS*, *SlZISO*, *SlZDS* and *SlCRTISO* [116]. Both SlFUL1 and SlFUL2 directly target *SlZISO*, *SlCRISO*, *SlSGR1*, *SlZEP*, *SlHYDB,* and *SlNCED*, with SlFUL1 additionally regulating *SlPSY1* and *SlGGPS2* [110]. In contrast, SlAP2c suppresses lycopene biosynthesis by inhibiting the transcription of *SlPSY1*, *SlZISO*, and *SlCRTISO* through promoter binding [117]. Some ripening-related TFs also exert indirect control through epigenetic or protein-interaction mechanisms. For instance, DNA methylation of Agamous-Like 1 (SlTAGL1) reduces *SlPSY1* expression and carotenoid production [118]. These layered regulatory networks underscore the tight coupling between ripening progression and carotenoid accumulation, a relationship that merits deeper exploration.

Beyond canonical ripening regulators, numerous other TFs contribute to carotenoid biosynthesis. Knockdown of *SlBEL11* produces orange fruits with elevated carotenoids, mediated by its direct repression of the *SlLCYB2* promoter [119]. SlMYB72 regulates not only chlorophyll biosynthetic genes but also carotenoid (*SlPSY1*, *SlZISO*, and *SlLCYB*) and flavonoid (*Sl4CL*, *SlCHS1*, and *SlCHS2*) biosynthetic genes [103]. SlZHD17 activates *SlPSY1*, *SlZISO*, and *SlSGR1* by binding to their promoters [104]. SlNAM2 and SlNAM3 enhance the expression of *SlZDS*, *SlPSY1*, and other ripening-associated genes, thereby coordinating ripening and quality traits [105]. SlNAC1 binds to the *SlPSY1* promoter and negatively regulates lycopene accumulation [120, 121], while SlNAC4 promotes ripening and carotenoid buildup likely through interactions with SlRIN and SlNOR [122]. Silencing of *SlNAC48* and *SlNAC19* results in orange fruits with reduced ethylene and lycopene levels and downregulated expression of carotenoid metabolism genes [123].

Light serves as the primary environmental signal regulating carotenoid biosynthesis in plants, and several light-responsive TFs have been demonstrated to directly target carotenogenic genes to modulate pigment accumulation in tomato fruit [47]. Phytochrome interacting factor 1a (SlPIF1a) binds to *SlPSY1* promoter and represses its expression [124]. Elongate Hypocotyl 5 (SlHY5) directly regulates the expression of *SlPSY1*, *SlZISO*, *SlCRTISO*, *SlLCYE*, *SlLCYB/SlCYCB*, and *SlVDE*. CRISPR-Cas9 mutants of *SlHY5* produce orange fruits with reduced carotenoid levels [125]. SlBBX20 activates *SlPSY1* by binding to its promoter [126]. Recent studies further indicate that both red and blue light influence chlorophyll and carotenoid metabolism through potential regulators such as SlbHLH93, SlERF4, SlWRKY20, SlARR11, SlNAC16, SlMYB12, SlbHLH46, and SlIAA29 [127, 128]. Nevertheless, the light-mediated regulatory network governing tomato fruit carotenoid biosynthesis remains incompletely understood and requires further exploration.

The most evident visual indicator of ripening is the color shift from green to red, concomitant with the transition of chloroplasts into chromoplasts. This process involves a synchronized reorganization of plastid membranes, marked by the disassembly of chlorophyll-containing thylakoids and the concurrent formation of carotenoid-rich membranous structures [28]. The transition is underpinned by a transcriptional reprogramming that represses photosynthesis-associated genes while activating those involved in carotenoid biosynthesis and sequestration [11]. Proteomic analyses reveal a global decline in photosynthetic proteins and a pronounced increase in non-photosynthetic plastid proteins dedicated to synthesis of carotenoids, fatty acids, and volatiles [129, 130]. Additionally, plastid transitions during ripening are facilitated by tomato SP1 homologs, which remodel the plastid protein import machinery to enable the proteome reorganization [131]. This transition is transcriptionally orchestrated by key ripening regulators, such as RIN and NOR, which directly activates carotenoid pathway genes and suppresses chlorophyll retention.

**Figure 5:**
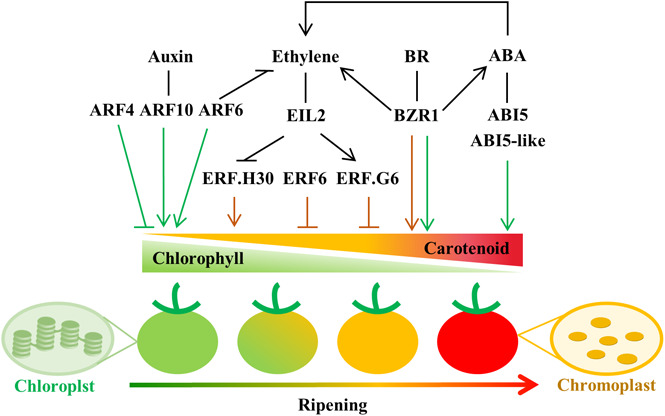
Key phytohormones and their associated TFs regulating chlorophyll and carotenoid metabolism during fruit ripening. As fruits ripen, chloroplasts differentiate into chromoplasts, leading to progressive chlorophyll degradation and substantial carotenoid accumulation. Phytohormones orchestrate this color transition by modulating downstream TFs, which in turn control the expression of genes involved in chlorophyll and carotenoid metabolism, thereby driving the visual color change. In the schematic, orange and green arrows indicate positive regulation of carotenoid biosynthesis and chlorophyll metabolism genes, respectively, by phytohormone-related TFs. Conversely, orange and green bar-headed lines denote negative regulation of carotenoid biosynthesis and chlorophyll metabolism genes, respectively, by phytohormone-related TFs. ABI5, ABA insensitive 5.

The chloroplast-to-chromoplast conversion is also coordinately regulated by phytohormones, with ethylene playing a central role (Fig. 5). As a climacteric fruit, tomato fruit ripening is initiated by ethylene signaling, which depends on key regulators, such as EIN3/EILs and ethylene response factors (ERFs), to influence carotenogenesis [132]. SlEIL2 reduces β-carotene accumulation in the late-ripening fruits by activating *SlERF·G6* and suppressing *SlERF·H30*, which in turn repress and promote *SlLCYB2* expression, respectively [133]. SlERF6 inhibits carotenogenesis by repressing *SlDXS* [134]. Unlike ethylene, auxin generally delays ripening. Its signaling components, SlARF2A and SlARF2B, contribute to carotenoid regulation [132]. However, whether these or other SlARFs directly target carotenoid biosynthesis genes remains unknown. Brassinosteroid (BR) levels rise during ripening and promote this process via SlBZR1, which activates ethylene biosynthesis genes and *SlPSY1* to enhance carotenoid accumulation [135]. SlBZR1 also enhances chilling tolerance of tomato plants by directly activating *SlNCED1* to promote ABA synthesis [136]. ABA plays a dual role in ripening and carotenogenesis in tomato fruit [137]. Acting upstream of ethylene biosynthesis, ABA synergizes with ethylene to modulate ripening progression [138]. Moreover, carotenoid accumulation correlates positively with ABA levels, as ABA transcriptionally activates key biosynthetic genes, such as *SlPSY1*, *SlPDS*, and *SlZDS*, in fruits [139, 140]. Beyond carotenoids, ABA orchestrates chlorophyll breakdown in leaves through direct binding of SlABI5 and SlABI5-like to the *SlSGRL* promoter [30]. Additionally, SL enhances carotenoid accumulation by stimulating ethylene production and elevating endogenous ABA [141]. Gibberellin levels peak at the mature green stage and decline during fruit ripening, opposing ethylene accumulation. It retards ripening by suppressing ethylene biosynthesis and signaling and downregulating *SlRIN*, *SlNOR*, and *SlCNR* [142, 143]. Jasmonic acid promotes lycopene accumulation in tomato fruits through an ethylene-independent pathway [144]. In summary, phytohormones are central regulators of tomato fruit ripening and carotenoid metabolism. Future research should prioritize elucidating the crosstalk between different hormonal pathways in modulating pigmentation, as well as identifying and characterizing hormone-associated transcription factors that directly target pigment metabolic genes (Fig. 5).

### Post-translational and epigenetic regulation of chlorophyll and carotenoid metabolism

While most studies on chlorophyll and carotenoid regulation focus on transcription, post-translational and epigenetic mechanisms also play important roles. For instance, UV-damaged DNA binding protein 1 (DDB1) and de-etiolated 1 (DET1) increase pigment content and chloroplast/chromoplast compartment size, underscoring their importance in plastid development [145, 146]. They form a Cullin 4 (CUL4)-based ubiquitin ligase complex that modulates SlGLK2 stability [147]. Similarly, the B-box (BBX) protein Constans-Like 1 (SlCOL1) interacts with SlBBX24 and stabilizes SlGLK2, promoting chlorophyll accumulation in immature fruits [148]. The UV-B photoreceptor SlUVR8 also stabilizes SlGLK2, acting as a positive regulator of chloroplast development in fruit [149]. SlSGR1 interacts with Balance of Chlorophyll Metabolism 1 (SlBCM1) and Yellow Green 1 (SlEGY1) to enhance its proteolytic processing and suppress chlorophyll degradation [150]. SlBCM1 additionally interacts with Genomes Uncoupled 4 (SlGUN4) to help maintain chlorophyll homeostasis in mature green and red fruits [150, 151]. SlSGR1 also interacts with SlPSY1 to inhibit its activity, thereby regulating lycopene and β-carotene accumulation [106]. SlPSY1 itself is subject to ubiquitination and degradation via Plastid Protein Sensing RING E3 ligase 1 (PPSR1) and the E2 enzyme SlUBC32, thereby influencing carotenoid biosynthesis in fruit [152]. Furthermore, SlCNR interacts with SlNOR to enhance the latter's transcriptional activation of carotenoid biosynthesis genes [116]. While such post-translational regulation largely affects regulators in pigment metabolism, the direct modification of metabolic enzymes remains poorly characterized and warrants further investigation.

Notably, chlorophyll and heme biosynthesis share common metabolic precursors (Fig. 1), and heme-derived products actively regulate chlorophyll production [153]. In tomato, the *yg-2* and *au* mutants display yellow-green leaves due to defects in heme oxygenase and phytochromobilin synthase, respectively [154]. Moreover, the heme-derived chromophore phytochromobilin interacts with GUN4, an activator of magnesium chelatase (MgCh), to enhance both the activity and stability of MgCh, effectively regulating chlorophyll biosynthesis through a mechanism that appears conserved across higher plants [155, 156]. This novel metabolite-protein regulatory paradigm reveals a previously overlooked layer of post-translational control that may also operate in tomato.

Epigenetic modifications also contribute to metabolic regulation of carotenoids. SlAP2c recruits co-repressors TOPLESS 2/4 and histone deacetylases (HDA1/3) to reduce histone acetylation at *SlPSY1*, *SlZISO*, and *SlCRTISO* genes and suppress their expression [117]. Other histone modifiers, such as the demethylase (JMJ6), heterochromatin protein 1b (LHP1), and histone variant H2A.Z, regulate carotenoid biosynthesis genes including *SlPSY1* and *SlVDE* [157–159]. Together, these findings illustrate the multi-layered control of pigment metabolism at transcriptional, post-translational, and epigenetic levels.

### Coordinated regulation of chlorophyll and carotenoid metabolism

The coordinated regulation of chlorophyll and carotenoid metabolism in fruit is crucial for fruit development, ripening, pigmentation, and nutritional quality. During ripening, plastids transition from chlorophyll-rich chloroplasts to carotenoid-rich chromoplasts, driving the characteristic color shift from green to red [28, 160]. This metabolic shift is accompanied by coordinated chlorophyll degradation and carotenoid accumulation and is orchestrated by plastid differentiation status, phytohormone signaling, and intracellular and environmental cues [161, 162]. Disrupting the balance between chlorophyll degradation and carotenoid biosynthesis can substantially impair ripening [163].

Several TFs have been shown to coordinately regulate both chlorophyll breakdown and carotenoid accumulation during ripening. In citrus, for example, CrWRKY42 and CrMADS3 simultaneously affect both pathways [164, 165]. Similarly, in tomato, key transcriptional regulators such as SlZHD17 and SlMYB72 directly target genes in both chlorophyll and carotenoid biosynthesis [103, 104]. Notably, canonical ripening regulators such as SlRIN and SlNOR activate *SlSGR1* to promote chlorophyll degradation while also binding to promoters of carotenogenic genes (e.g., *SlPSY1*, *SlPDS*) to synchronize pigment turnover [107, 109]. This tightly integrated transcriptional network highlights the sophisticated metabolic crosstalk that underlies fruit pigmentation, emphasizing its critical role in both visual appearance and nutritional quality.

## Conclusions and future perspectives

Decades of research have significantly advanced our understanding of chlorophyll and carotenoid metabolism, regulation, and their roles in tomato fruit quality. However, numerous questions necessitate further investigation. First, the specific dephytylase(s) responsible for chlorophyll dephytylation during fruit ripening have yet to be definitively identified, limiting a complete understanding of chlorophyll catabolism. Second, while CCDs and NCEDs are known to maintain carotenoid homeostasis and generate flavor-related apocarotenoid volatiles, their full functional versatility and regulatory networks require further elucidation to optimize both pigment accumulation and fruit flavor. Third, genetically engineering the carotenoid pathway in tomato to produce high-value compounds like bixin or capsanthin remains a promising strategy for enhancing fruit color and nutritional quality. Additionally, the regulation of pigment metabolism by shared TFs underscores the intricate link between color development and broader metabolic networks. Identifying key co-regulators within these networks is essential to decipher the mechanisms driving fruit color transitions. Moreover, phytohormones and environmental signals (e.g., light, temperature) converge to modulate pigment metabolism. A systems-level understanding of their crosstalk will provide insights into the plasticity of fruit pigmentation under varying conditions. Finally, despite its established importance in regulating protein function, the post-translational modifications (e.g. phosphorylation, ubiquitination, and lysine acetylation) of core pigment metabolic enzymes remain significantly underexplored and constitutes a vital research frontier. These efforts will deepen our understanding of the regulatory architecture controlling pigment metabolism, facilitating the development of tomato varieties with superior visual and nutritional traits.
